# Brainstem Auditory Physiology in Children with Listening Difficulties

**DOI:** 10.1016/j.heares.2023.108705

**Published:** 2023-01-20

**Authors:** Lisa L. Hunter, Chelsea M. Blankenship, Barbara Shinn-Cunningham, Linda Hood, Lina Motlagh Zadeh, David R. Moore

**Affiliations:** 1 Communication Sciences Research Center, Cincinnati Children’s Hospital Medical Center, Cincinnati, Ohio. USA.; 2 Research in Patient Services, Cincinnati Children’s Hospital Medical Center, Cincinnati, Ohio. USA.; 3 College of Medicine, Otolaryngology, Communication Sciences and Disorders, University of Cincinnati, Cincinnati, Ohio. USA.; 4 College of Allied Health Sciences, University of Cincinnati, Cincinnati, Ohio. USA.; 5 Neuroscience Institute, Carnegie Mellon University, Pittsburgh, PA. USA.; 6 Vanderbilt University Medical Center, Nashville, TN. USA.; 7 Manchester Centre for Audiology and Deafness, University of Manchester, U.K.

**Keywords:** Listening difficulty, auditory processing disorder, middle ear muscle reflex, auditory brainstem response, speech in noise, extended high frequency

## Abstract

Children who have listening difficulties (LiD) despite having normal audiometry are often diagnosed as having an auditory processing disorder. A lack of evidence regarding involvement of specific auditory mechanisms has limited development of effective treatments for these children. Here, we examined electrophysiologic evidence for brainstem pathway mechanisms in children with and without defined LiD. We undertook a prospective controlled study of 132 children aged 6–14 years with normal pure tone audiometry, grouped into LiD (N=63) or Typically Developing (TD; N=69) based on scores on the Evaluation of Children’s Listening and Processing Skills (ECLiPS), a validated caregiver report. The groups were matched on age at test, sex, race, and ethnicity. Neither group had diagnoses of major neurologic disorder, intellectual disability, or brain injuries. Both groups received a test battery including a measure of receptive speech perception against distractor speech, Listening in Spatialized Noise - Sentences (LiSN-S), along with multiple neurophysiologic measures that tap afferent and efferent auditory subcortical pathways. Group analysis showed that participants with LiD performed significantly more poorly on all subtests of the LiSN-S. The LiD group had significantly greater wideband middle ear muscle reflex (MEMR) growth functions in the left ear, and shorter Wave III and Wave V latencies in auditory brainstem responses (ABR). Across individual participants, shorter latency ABR Wave V correlated significantly with poorer parent report of LiD (ECLiPS composite). Greater MEMR growth functions also correlated with poorer ECLiPS scores and reduced LiSN-S talker advantage. The LiD and TD groups had equivalent summating potentials, compound action potentials, envelope-following responses, and binaurally activated medial olivocochlear reflexes. In conclusion, there was no evidence for auditory synaptopathy for LiD. Evidence for brainstem differences in the LiD group was interpreted as increased efferent control or central gain, with shorter ABR Wave III and V latencies and steeper MEMR growth curves. These differences were related to poorer parent report and speech perception in competing speech ability.

## 1.0 INTRODUCTION

Children with difficulty understanding and responding to speech in challenging listening conditions often present to audiology clinics for diagnosis and treatment, and if they have normal pure tone sensitivity, the source of their problems and how to treat them is unclear. We have adopted the term LiD, defined by validated caregiver report, to categorize such children in an NIH-funded prospective longitudinal study, and recently reported that the peripheral auditory system was overall functioning normally for the group with LiD, except for a subgroup with a history of otitis media who had poorer extended high frequency (EHF) hearing (Hunter et al., 2021). We also reported on a wide range of auditory and cognitive measures in the same group, with the primary result that supramodal cognitive factors (e.g. language, memory and attention) were highly correlated with their reported listening difficulties, consistent with many previous studies in other groups of children with LiD (Petley et al., 2021).

Traditionally, children with LiD who perform poorly on tests of speech and non-speech auditory perception may be diagnosed as having auditory processing disorder (APD), a poorly understood, heterogenous condition that often co-exists with developmental language disorder and attention deficit hyperactivity disorder (Dawes & Bishop, 2009; Ferguson, Hall, Riley, & Moore, 2011; Miller & Wagstaff, 2011; Moore et al., 2018; Sharma, Purdy, & Kelly, 2009). The label APD has thus been questioned as a distinct clinical entity (Kamhi, 2011; Miller, 2011; Moore, 2018; Vermiglio, 2014) and behavioral auditory processing tests produce highly variable results even when published clinical guidelines are used (Jutras et al., 2007; Wilson and Arnott, 2013). Alternatively, neurophysiologic techniques have the advantage of identifying diverse underlying mechanisms from the periphery to the central auditory nervous system, including ascending and descending pathways, without relying upon behavioral responses. They have thus been recommended to evaluate auditory pathway involvement in APD (AAA, 2010; Ankmnal-Veeranna, Allan, & Allen, 2019; ASHA, 2005; Jerger & Musiek, 2000). However, studies using these techniques have produced inconsistent findings, probably due to varying definitions of APD, retrospective design, inadequate methodologic control, small group sizes, or lack of a control group (Liu, Zhu, Chen, Hong, & Chi, 2021).

Ascending auditory neuronal transmission can be investigated from the inner hair cells to the lateral lemniscus using electrocochleographic (ECochG) and auditory brainstem responses (ABR). The ABR is a diagnostic neurophysiologic measure, that is highly reliable with low intrasubject variability when controlled for sex, as females generally have shorter latencies than males (McClelland & McCrea, 1979). Developmental trajectories reflect myelination and provide an indirect measure of brainstem processing time (Hecox & Burkard, 1982; Salamy, 1984). The difference between Wave V and Wave I is thought to reflect central conduction time, and has been found to be extended in children with autism (Miron et al., 2021; Rosenhall, Nordin, Brantberg, & Gillberg, 2003; Thivierge, Bedard, Cote, & Maziade, 1990; Wong & Wong, 1991).

Temporal encoding has been investigated using narrow- (Bharadwaj, Verhulst, Shaheen, Liberman, & Shinn-Cunningham, 2014) and multi-band (Wang, Bharadwaj, & Shinn-Cunningham, 2019) complex tones to measure envelope following responses (EFR) driven by specific frequency regions of the cochlea. These responses have been proposed as a neurophysiological measure for detecting subtle temporal coding deficits in listeners with normal hearing who have difficulty understanding target sounds in complex acoustic scenes, such as might arise with cochlear synaptopathy (Encina-Llamas, Harte, Dau, Shinn-Cunningham, & Epp, 2019; Shaheen, Valero, & Liberman, 2015). Deficits in spectrotemporal processing have been reported in children with APD and are associated with poorer speech in noise perception (Ankmnal Veeranna, Allan, Macpherson, & Allen, 2019; Lotfi, Moossavi, Afshari, Bakhshi, & Sadjedi, 2020), although such deficits also present in visual temporal processing, so may reflect general processing speed problems (Dawes et al., 2009). Thus far, EFR has not been investigated in children with LiD and could shed light on neural mechanisms of auditory temporal processing in children with LiD.

Multimodal pathways, deriving from anterior temporal, and inferior frontal and parietal cortex interact with ascending processing in the auditory cortex, giving rise to a large descending auditory system (Hackett, 2011; Moore, Rosen, Bamiou, Campbell, & Sirimanna, 2013). One important function of such cortical and sub-cortical efferent pathways is to modulate ascending sensory auditory information from the ear. Recent evidence from animal studies suggests that top-down pathways to subcortical auditory nuclei (medial geniculate and inferior colliculus) are influential in challenging listening situations (Blackwell, Lesicko, Rao, De Biasi, & Geffen, 2020; Souffi, Nodal, Bajo, & Edeline, 2021). Our striking human ability to maintain speech understanding in highly degraded listening conditions of noise, reverberation, and competing meaningful speech (the “cocktail party” effect) depends in part on these efferent cortical pathways, which modulate neural activity in the inferior colliculus (Hernandez-Perez et al., 2021).

The auditory efferent system can be assessed with the medial olivocochlear reflex (MOCR) and middle ear muscle reflexes (MEMR), the two major descending systems that modify the auditory periphery to enhance speech in noise perception (Liberman & Guinan, 1998; Mukerji, Windsor, & Lee, 2010). The MEMR pathway begins in the auditory periphery (middle ear cochlea, auditory nerve) and projects to the cochlear nucleus in the brainstem, the first ascending central auditory relay station. From there, ventral cochlear nucleus interneurons activate efferent stapedius motoneurons and initiate a muscle contraction in the same ear (ipsilateral) and, also in the contralateral ear, as the reflex is consensual, like the pupillary reflex (Mukerji et al., 2010). Thus, the MEMR includes both ascending afferent pathways and descending efferent pathways to the same and opposite ears. The MEMR is primarily active in low frequencies and has three known essential functions. The MEMR minimizes masking of speech frequencies by intense background noise to preserve speech perception in noise, thus is relevant to mechanisms for listening difficulties (Mahoney, Vernon, & Meikle, 1979). In addition, it offers some protection to the ear during high intensity sounds (Zakrisson, Borg, Liden, & Nilsson, 1980) and it reduces the level of one’s own voice by activating just prior to vocalization (Borg & Zakrisson, 1975).

Children with APD diagnosed with auditory processing tests exhibited poorer contralateral acoustic reflex thresholds at 500, 1000 and 2000 Hz compared to typical children (Saxena, Allan, & Allen, 2017) but MEMR thresholds were not different in children with suspected APD compared to those diagnosed with APD. This finding was consistent with an earlier study that found no difference in MEMR thresholds based on diagnosed versus suspected APD (Allen & Allan, 2014). In a study of 66 children with suspected APD compared to 30 typically developing children, MEMR growth as a function of stimulus intensity was found to be lower for contralateral (but not ipsilateral) reflex growth at 500, 1000 and 2000 Hz in children diagnosed or suspected with APD compared to typically developing children (Saxena, Allan, & Allen, 2015). Using a wideband absorbance technique measured with real-ear recording to account for ear canal volume, we recently reported that children with LiD showed no differences in ipsilateral or contralateral MEMR thresholds compared to a typically developing control group (Hunter et al., 2021).

In contrast to these findings of shallower MEMR growth in children with suspected or diagnosed APD, patients exposed to aminoglycoside antibiotics had greater MEMR growth than non-exposed controls, despite having normal pure tone thresholds (Westman, Putterman, Garinis, Hunter, & Feeney, 2021). The steeper growth was interpreted as evidence for central gain, consistent with animal and temporal bone studies showing synaptic damage with aminoglycoside exposure. The MEMR is highly plastic to both auditory deprivation and acoustic stimulation, showing threshold decreases with earplugging and increases in control ears after seven days (Munro, Turtle, & Schaette, 2014.). Interestingly, Munro and colleagues also found that MEMR threshold decreases were accompanied by bilateral loudness judgment changes.

MOCR strength has been quantified using transient-evoked otoacoustic emissions (TEOAEs) in quiet and with monaural and binaural elicitors in a forward masking paradigm (Berlin, Hood, Hurley, Wen, & Kemp, 1995). MOCR dysfunction has been suggested to affect speech perception due to longer cochlear ringing and increases in forward masking (Boothalingam, Allan, Allen, & Purcell, 2015). The MOCR may be selectively activated by distorted (e.g., vocoded) speech rather than by clear speech in noise (Hernandez-Perez et al., 2021). Several studies have examined MOCR in children with suspected APD or LiD, but results have been conflicting. An evidence-based review of nine such studies in children with APD, dyslexia, learning impairment, or specific language impairment showed that the MOCR was reduced in DPOAE or TEOAE suppression relative to controls in 4 studies, while 3 studies showed no significant group differences (Mishra, 2014). Variability across studies is likely due to subject selection, methodological differences and test-retest variability (Boothalingam, Allan, Allen, & Purcell, 2019; Mertes & Goodman, 2016; Mertes & Leek, 2016), inadequate control of OAE signal-to-noise ratio (SNR), attention effects (Mishra, 2014), or unintended activation of the MEMR (Marks & Siegel, 2017; Mishra, 2014).

To examine mechanisms that may underly LiD, we conducted a prospective evaluation of afferent and efferent auditory brainstem function in children with and without defined LiD using neurophysiologic tests selected to investigate specific regions of the auditory pathway. We hypothesized that children with LiD have deficiencies in either ascending or descending auditory brainstem pathways that relate to their speech-in-noise deficits.

## 2.0 MATERIALS AND METHODS

### 2.1 Participants

The study was approved by the Cincinnati Children’s Hospital (CCH) Institutional Review Board. Parental permission and child assent for those 11 years and older was obtained before assessments. This report is part of a broader longitudinal Sensitive Indices of Childhood Listening Difficulty (SICLiD) study that aimed to uncover mechanisms of LiD. Participants with LiD were initially recruited from a medical record review of over 1,100 children assessed for suspected Auditory Processing Disorder (APD) at Cincinnati Children’s Hospital. In that sample, 179 were diagnosed by an audiologist as having APD and 364 as having an auditory processing weakness (Moore et al., 2018). Caregivers of the children diagnosed with APD or weakness who responded to an invitation to participate in this study were sent three in depth medical, educational, and listening difficulty questionnaires to determine eligibility. Recruitment was also done through print, electronic, social, and digital media at hospital locations and in the local and regional area seeking children with listening difficulties. Typically developing children were recruited from print, electronic, social, and digital media seeking children with no hearing or listening concerns. Parents of all children were asked to fill out the same background questionnaires. Children with LiD were aged 6 to 14 years old at enrollment and were age- and gender-matched to typically developing (TD) children by proportional sampling. The LiD group was defined based on low (< −1 s.d.) age-standardized total scores on the Evaluation of Children’s Listening and Processing Skills (ECLiPS), a validated parent questionnaire (J.G. Barry & D.R. Moore, 2021; Barry, Tomlin, Moore, & Dillon, 2015). The ECLiPS is a Likert-scaled 38-item parent questionnaire that is specifically designed to identify listening and other difficulties associated with APD. ECLiPS questions were developed based on responses in focus groups by parents regarding concerns about their children who were referred by audiologists for APD assessment. It was developed by applying a systematic procedure outlined by Cronbach & Meehl (1955) requiring (1) specification of the psychological construct (latent traits to be measured), (2) development of a sufficiently broad ranging but carefully worded item pool, and (3) assessment of the construct validity and reliability of measurement of the final scale. The British Society of Audiology (2011) position statement for APD provided the basis for specifying the psychological construct for the ECLiPS. All subscales of the ECLiPS have high test-retest reliability, with intra-class correlations (ICCs) above 0.8 (Barry & Moore, 2021). Construct validity has been demonstrated through convergence with other established tests that measure similar skills, including the Children’s Auditory Processing Performance Scale (CHAPS), the Listening Inventory for Education (LIFE), the Teacher’s Evaluation of Auditory Performance (TEAP), the Fisher’s Auditory Problems Checklist (FAPC), and also with tests of APD, specifically the Dichotic Digits test and the Frequency Pattern Test (Barry et al., 2015).

Inclusion criteria for both groups were no major neurologic or cognitive diagnoses, no brain injuries, normal bilateral standard frequency pure tone hearing thresholds (≤20 dB HL; .25–8 kHz), normal otoscopy, and normal 226-Hz tympanometry (tympanometric width < 250 daPa). Study data were collected and managed using Research Electronic Data Capture (REDCap) electronic data capture tools hosted at the University of Cincinnati (Harris et al., 2019; Harris et al., 2009).

### 2.2 Procedures

#### Audiological Assessment

Otoscopy was completed and if necessary, cerumen was removed before audiometry. All audiometric tests were completed in a double-walled soundproof booth (Industrial Acoustics Company, North Aurora, IL) that meets standards for acceptable room noise for audiometric rooms (ANSI/ASA 1999 (R2018). Standard (0.25 to 8 kHz) and EHF (10–16 kHz) thresholds were measured using the manual Hughson–Westlake method for the range of 0.25 to 8 kHz at octave intervals and at four additional frequencies (10, 12.5, 14, and 16 kHz) using an Equinox audiometer (Interacoustics Inc., Middlefart, Denmark) with Sennheiser HDA-300 circumaural earphones (Old Lyme, CT). Calibration was completed according to ISO 389.9 (International Organization for Standardization, 2004) for standard frequencies and ISO 389–1 (International Organization for Standardization, 2017) for EHF. Normal hearing was defined as thresholds ≤ 20 dB HL (0.25–8 kHz). If air conduction thresholds were greater than 20 dB HL, bone conduction thresholds were measured between 0.5 and 4 kHz using appropriate narrowband masking in the contralateral ear (Radioear Inc. B-71 bone vibrator, New Eagle, PA) to determine the type of hearing loss.

#### Speech in Noise

The Listening in Spatialized Noise – Sentences (LiSN-S) task, North American version (Brown, Cameron, Martin, Watson, & Dillon, 2010; Cameron & Dillon, 2007) was administered using a laptop, a task-specific soundcard, and Sennheiser HD 215 headphones. Participants were asked to repeat a series of target sentences, presented from directly in front (0°), while ignoring two distracting sentences. There are four listening conditions, in which the distractors change voice (either different or the same as target) and/or position (either both at 0° or at −90° and +90° degrees relative to the listener). The test is adaptive; the level of the target speaker decreases or increases in SNR depending on listener accuracy. Each condition continues for 22–30 trials, ending when the standard error of reversals is < 1 dB. The 50% correct SNR is calculated for the ’Low cue speech reception threshold’ (SRT; same voice, 0° relative to the listener) and the ’High cue SRT’ (different voice, 90° degrees relative to the listener). Three ’derived scores’ are the Talker Advantage (difference in thresholds for different voice vs. same voice when the distractors are at 0˚), Spatial Advantage (difference in thresholds for spatially separated vs. spatially collocated distractors when the voice is the same), and Total Advantage (difference in the High cue SRT and the Low cue SRT).

#### Middle Ear Muscle Reflex

Wideband tympanometry was performed prior to MEMRs (acoustic absorbance and group delay) using click stimuli (bandwidth 0.2 to 8 kHz) delivered while ear canal pressure was swept from +200 to −400 daPa using a custom recording system (Keefe, Feeney, Hunter, & Fitzpatrick, 2017) coupled to an AT235 immittance system (Interacoustics Inc., Middlefart, Denmark). MEMRs were measured using the wideband absorbance technique with custom MATLAB software described by Keefe et al. (2017). The probe assembly contained a high bandwidth receiver that delivered wideband clicks as the probe stimulus, and a second receiver with the same bandwidth that allowed higher stimulus levels. Broadband noise (BBN, 0.2 to 8 kHz) and pure-tone stimuli (0.5, 1, and 2kHz) were presented ipsilaterally and contralaterally to the probe ear while the click stimulus was presented ipsilaterally to measure absorbance changes in the ear with the microphone. Ear canal air pressure was adjusted to the average peak tympanometric pressure obtained for down swept and upswept wideband tympanometry. To record MEMR responses, probe clicks were averaged across four stimuli, calibrated in a 2-cc coupler and in the real ear. Contralateral and ipsilateral MEMR testing used response averaging, artifact rejection and signal processing techniques to measure threshold, onset latency, and amplitude growth with click level. Amplitude growth of the MEMR was recorded at 10 levels (L1-L10, where L1 is the lowest level). For BBN, MEMRs were measured at 0 dB SPL and then from 50 to 90 dB SPL in 5 dB steps. Similarly, for pure-tones, MEMRs were measured at 0 dB SPL and then 65 to 105 dB SPL in 5 dB steps. At each stimulus level, the MEMR shift was measured in cumulative weighted absorbed power level in dB, averaged across lower frequencies (0.2 to 2.4 kHz) where the reflex is primarily active.

#### Electrocochleography and Auditory Brainstem Response

**Figure 1.**
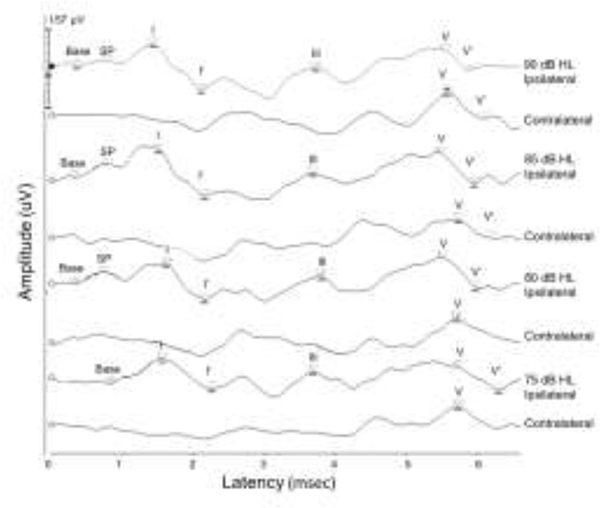
Ipsilateral and contralateral ABR waveforms illustrating template marking of peak latency and amplitudes for a representative waveform at intensities from 75 to 90 dB nHL in 5 dB steps. Base is marked at the start of the rise prior to the SP to measure amplitude. I’ and V’ are marked at the trough following Waves III and V to measure amplitudes.

Combined ECochG and ABR recordings were obtained using the Intelligent Hearing Systems (IHS, Miami, FL) SmartEP two-channel system with the universal SmartBox platform. IHS ultra-shielded insert earphones (300Ω) coupled to gold leaf tiptrodes were used to deliver stimuli and simultaneously to serve as the negative electrodes. All recordings were performed in a double-walled sound booth. Stimuli were alternating clicks, split into rarefaction and condensation buffers at 75, 80, 85 and 90 dB nHL, at a rate of 11.1 clicks/sec, with 2048 sweeps recorded per intensity, and repeated for a total of 4096 sweeps per intensity. Filters were 0.1–3 kHz with gain of 100k. The two-channel recording montage was high forehead (positive) to bilateral ear canals, with the ground at the low forehead. Care was taken to insert the tiptrodes as deeply as possible without disturbing the gold foil. Impedances were maintained at less than 5 kOhms, and <2 kOhms difference between electrodes. If peaks were not easily discernable or the number of artifacts was greater than or equal to the 10% of the number of sweeps, the recording was repeated, and the best two recordings (best defined peaks with least noise) were analyzed. Ipsilateral and contralateral recordings were analyzed using a normative latency/intensity template to guide selection for consistent marking of latency and amplitude of the summating potential (SP; base to shoulder), and Waves I, III and V (peak to the negative trough following the waveform), as in Figure 1. Recordings were manually marked with blinding of subject information by one investigator and were independently cross-checked by a blinded second scorer to ensure agreement on marked latencies and amplitudes. Any discrepancies were reviewed by the first author (LLH) with blinding maintained. Twenty-one participants did not complete ABR testing due to insufficient time or scheduling issues, and seventeen participants were excluded due to inadequate ABR quality (9 LiD, 8 TD).

#### Binaural Medial Olivocochlear Reflex

The MOCR was measured using TEOAEs with an Intelligent Hearing Systems dual channel universal SmartBox (SmartTrOAE, Miami, FL) with two matched OAE 10D Probes designed to present synchronized binaural suppression noise (elicitor). All recordings were performed in a double-walled sound booth. TEOAEs were first recorded in quiet with non-linear rectangular clicks (three clicks of positive polarity followed by a fourth click of inverse polarity with a relative magnitude of 9.5 dB higher than the corresponding positive clicks) in each ear (75 μsec, 80 dB peSPL, 21.1 per sec). To ensure that baseline responses were present, we required 3 dB SNR for three or more frequency bands out of 6, and >60% whole wave reproducibility, and artifacts were required to be below 10% of the total. The MOCR was then elicited by recording TEOAEs with clicks (75 μsec, 60 dB peSPL, 21.1 per sec) with 256 sweeps in each condition. The 60 dB peSPL TEOAE activator and the suppression elicitor (60 dB SPL white noise presented binaurally) were set to be below MEMR thresholds to minimize activating the stapedial reflex during MOCR measurement. The activator and elicitor stimuli were interleaved with order of testing as follows: Quiet Condition 1; Binaural Elicitor Condition 1; Quiet Condition 2; Binaural Elicitor Condition 2. The elicitor was presented in a forward masking paradigm, with a 400 ms. duration elicitor, and an interstimulus interval of 10 ms. If the SNR was <3 dB or the correlation was < .6, then an additional 256 sweeps were recorded (512 total). Artifacts were required to be less than 10% of the total. A reclining chair was used to encourage participants to be still and quiet. Attention was controlled by having the subject watch a silent video with captions. If the recording did not meet SNR criteria, the probe was refit, and the participant was reminded to remain quiet and still. Recordings were analyzed using the IHS MOCR analysis module set to a 10 ms. window from 8 to 18 ms., Hanning filter, 2 ms. resolution, and coherence display setting. The RMS amplitude for each waveform was recorded, and the binaural elicitor condition was subtracted from the quiet condition to obtain the average MOCR strength estimate for each ear.

#### Envelope Following Response

The EFR was recorded using a multi-channel actiCHamp Brain Products system (Brain Products GmbH, Inc., Munich, Germany). A 64-electrode cap was placed on the scalp with electrodes placed at equidistant locations. This “infracerebral” cap covers a larger area than is typical in a 10–20 system (Hine & Debener, 2007). The reference channel was located at vertex (Cz) while the ground electrode was located on the midline, 50% of the distance to nasion. Responses were recorded using a sampling rate of 5 kHz. The stimuli were transposed tones generated offline in MATLAB (Natick, MA) and stored for playback with a sampling rate of 48.8 kHz. Stimuli were presented at three different modulation depths, 100%, 63% and 40%, with a modulation rate of 100 Hz and carrier frequency of 4 kHz (Bharadwaj et al., 2014). The stimuli were 400 ms. in duration and 1000 trials of each modulation depth were recorded. The inter-trial interval was jittered between 410 and 510 ms. to ensure that EEG noise (not in response to the stimulus) occurs at a random phase between −π and π for frequencies above 10 Hz. Stimuli were presented diotically over ER-2 insert earphones (Etymotic Research, Elk Grove Village, IL) with the level at each ear at 70 dB SPL. All recordings were performed in a Faraday shielded double-walled sound booth. Participants were encouraged to sleep during testing, which took approximately 45 minutes to complete.

Electrophysiological data were analyzed using Brain Vision Analyzer ver. 2.0 (Brain Products GmbH, Inc., Munich, Germany) and custom Python and MATLAB scripts. To minimize signal contributions from cortical sources before epoching and to remove 60 Hz line noise, data were re-referenced to the left and right average mastoid reference and high-pass filtered in MATLAB with a 70 Hz cutoff frequency using an FIR filter with zero group-delay (Herdman et al., 2002; Kuwada et al., 2002). Response epochs from −50 to 250 ms (relative to the stimulus onset time of each trial) were segmented out from each channel. Epochs with signals whose dynamic range exceeded 100 μV in any channel were excluded from further analysis to remove movement and muscle activity artifacts. Principle component analysis was used to combine channels and reduce recording time. See Bharadwaj and Shinn-Cunningham (2014) for further details.

#### Statistical Analysis

For all measures, each recording was monitored online for excessive artifacts and noise and was repeated if necessary, during the same session, after taking care to obtain the quietest condition and best probe fit and/or electrode connection possible. Data were exported for each test, then were further analyzed for recording artifacts. If the test was repeated, the best quality recordings (lowest noise and artifacts) were selected for further analysis.

Statistical analysis was completed using JASP version 0.13.1 (University of Amsterdam). Results were examined initially with descriptive statistics to summarize sample demographics and outcome measurements. Interval variables were summarized by central tendency and dispersion, and categorical variables were described by frequencies and percentages. Two-sample t-tests and Chi-Square tests were used to compare the demographics between the children with LiD and TD. Boxplots were created to study the distribution of the outcomes. Outcome variables were analyzed first in univariate, then multivariate mixed models that included group (TD or LiD), age at test, sex, PE tube history, and EHF hearing loss (EHFHL) as independent factors. Holm multiple adjustment was applied for pairwise comparisons among the levels of the significant factors to maintain the experiment-wise error rate below alpha = .05 (Staffa & Zurakowski, 2020). Multivariate forward stepwise linear regressions were calculated to explore the relationship among the electrophysiologic and behavioral outcomes. P value <0.05 was required for entry to the model, and p >0.10 for removal.

## 3.0 RESULTS

### 3.1 Demographics

The current report includes 132 participants (TD=69, LiD=63) from the first SICLiD longitudinal evaluation (Table 1). There were no significant group differences in age at test, sex, race, ethnicity, or history of pressure equalization (PE) tube insertion to treat otitis media. The LiD group had significantly lower maternal education levels, more EHF hearing loss, and poorer scores on the ECLiPS (by selection) and LiSN-S, compared to the TD group.

### 3.2 Electrocochleography and Auditory Brainstem Response

**Figure 2.**
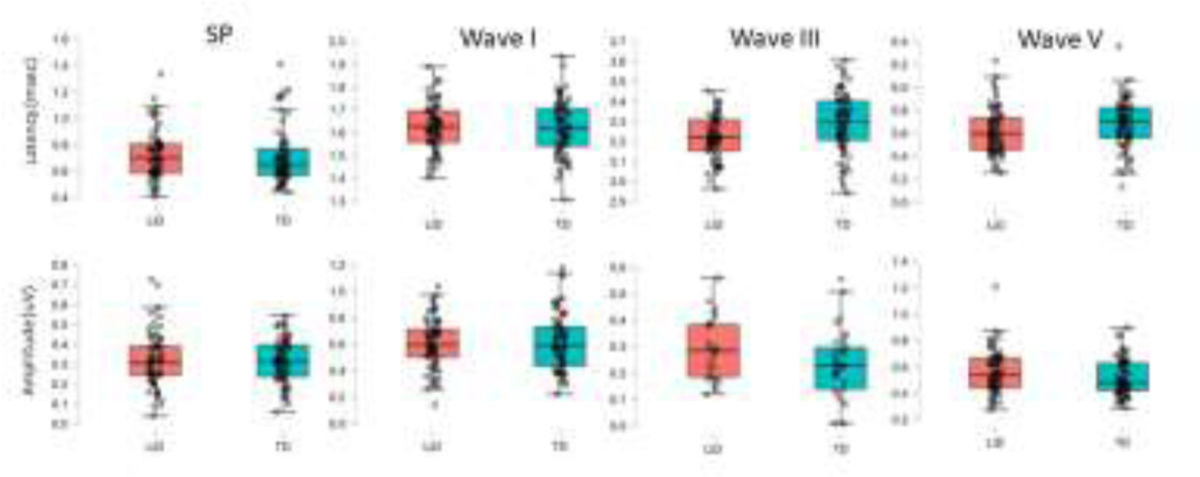
Top row: Box and scatterplots for ipsilateral ABR latency for the summating potential (SP), Waves I, III and V, averaged across intensity measured at 75, 80, 95 and 90 dB nHL re: normal adult threshold for clicks. The LiD (pink) and TD group (blue) groups are plotted separately, with the median center line, interquartile range (box), and 95% range (stems). Individual ears are shown as open circles. in dB HL. Bottom row: Amplitude of ABR waveforms is plotted in the same format as described above. N= 67 ears for the TD group and 66 ears for the LiD group.

**Figure 3.**
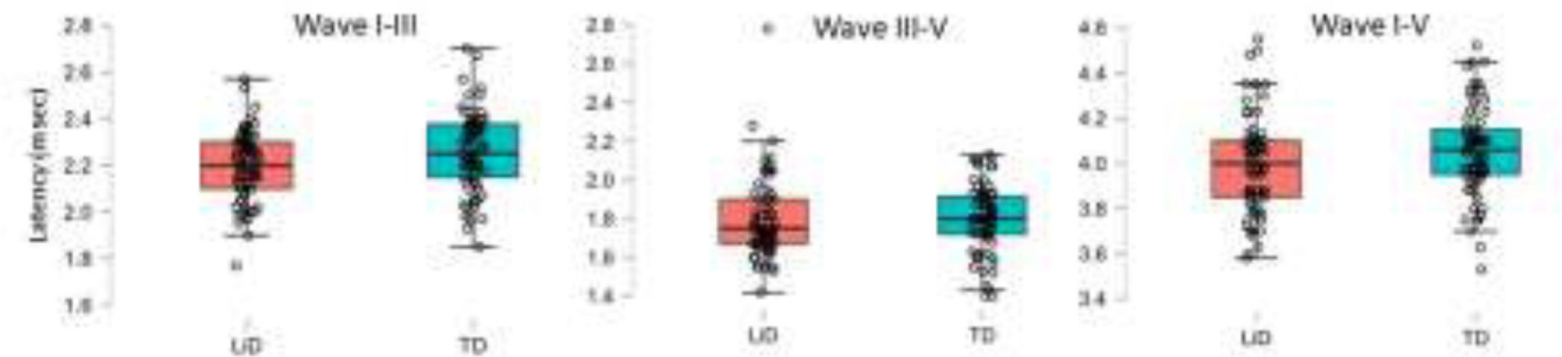
Box and scatterplots for ipsilateral ABR interpeak latencies for Waves I-III, III-V, and I-V at 90 dB nHL. The LiD (pink) and TD group (blue) groups are plotted separately, with the median center line, interquartile range (box), and 95% range (stems). Individual ears are shown as open circles. N= 67 ears for the TD group and 66 ears for the LiD group. Overall ANOVA: Group p=0.089, Ear p=0.357.

**Figure 4.**
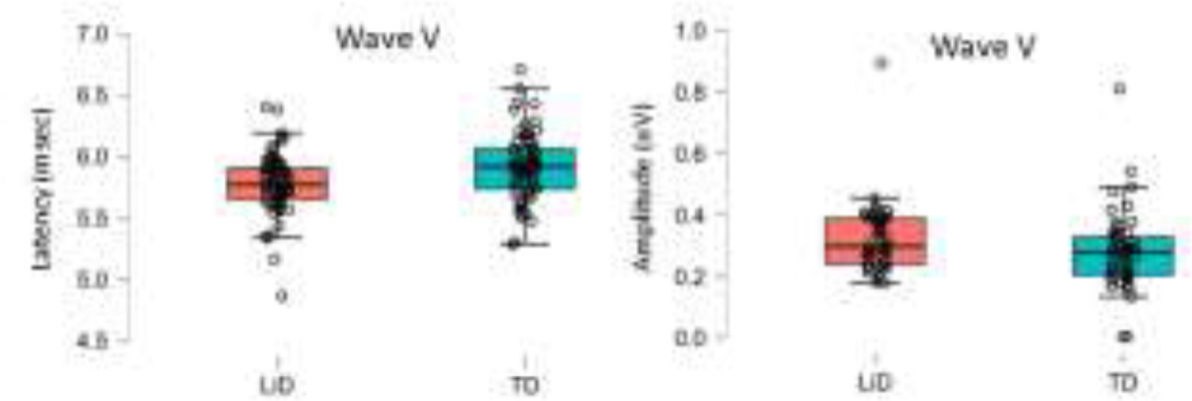
Contralateral ABR latency (left panel) and amplitude (right panel) Wave V averaged across intensity measured at 75, 80, 95 and 90 dB HL re: normal adult threshold for clicks. The LiD (pink) and TD group (blue) groups are plotted separately with the median center line, interquartile range (box), and 95% range (stems). Individual ears are shown as open circles. N= 67 ears for the TD group and 66 ears for the LiD group.

ABR responses were obtained from 37 LiD participants (N=67 ears) and 43 TD participants (N=66 ears). Ipsilateral and contralateral ABR latency and amplitude growth functions as the stimulus increased were examined with separate RMANOVAs. Stimulus intensity was the repeated factor, group and ear were between subject factors, while age, sex, average EHF hearing levels (10–16 kHz) and tube history were included as covariates. Results for ABR latency and amplitude for ipsilateral and contralateral waveforms, and ipsilateral interpeak latencies are shown in Figures 2–4 with RMANOVA results in Table 2. There were significant decreases, as expected, in ipsilateral ABR latency with increasing stimulus level for SP, Wave I and Wave V, as well as amplitude of SP and Wave I. Peripheral waveforms generated by the cochlea (SP) and auditory nerve (Wave I) had similar latency and amplitude between groups. The LiD group had significantly shorter ipsilateral Wave III and Wave V latencies than the TD group (Figure 2; C, D, G, H; Table 2). The effect sizes were small to medium (Wave III: ƞ^2^_p_ = 0.091; Wave V: ƞ^2^_p_ = 0.04). The left ear had significantly shorter latencies than the right ear for SP and Wave I. There were no significant differences between groups for amplitude of any waveforms. The ipsilateral Wave V to Wave I amplitude ratio was not significantly different between groups and no group difference in interpeak latencies were found for Wave I-III, III-V, and I-V; Figure 3, Table 2. There were differences in interpeak intervals, but they did not reach statistical significance when corrected for multiple comparisons, probably because variability of the subtracted measure (interpeak interval) is higher than absolute latency. Lastly, there were no significant group differences in contralateral Wave V latency or amplitude (Figure 4).

**Figure 5.**
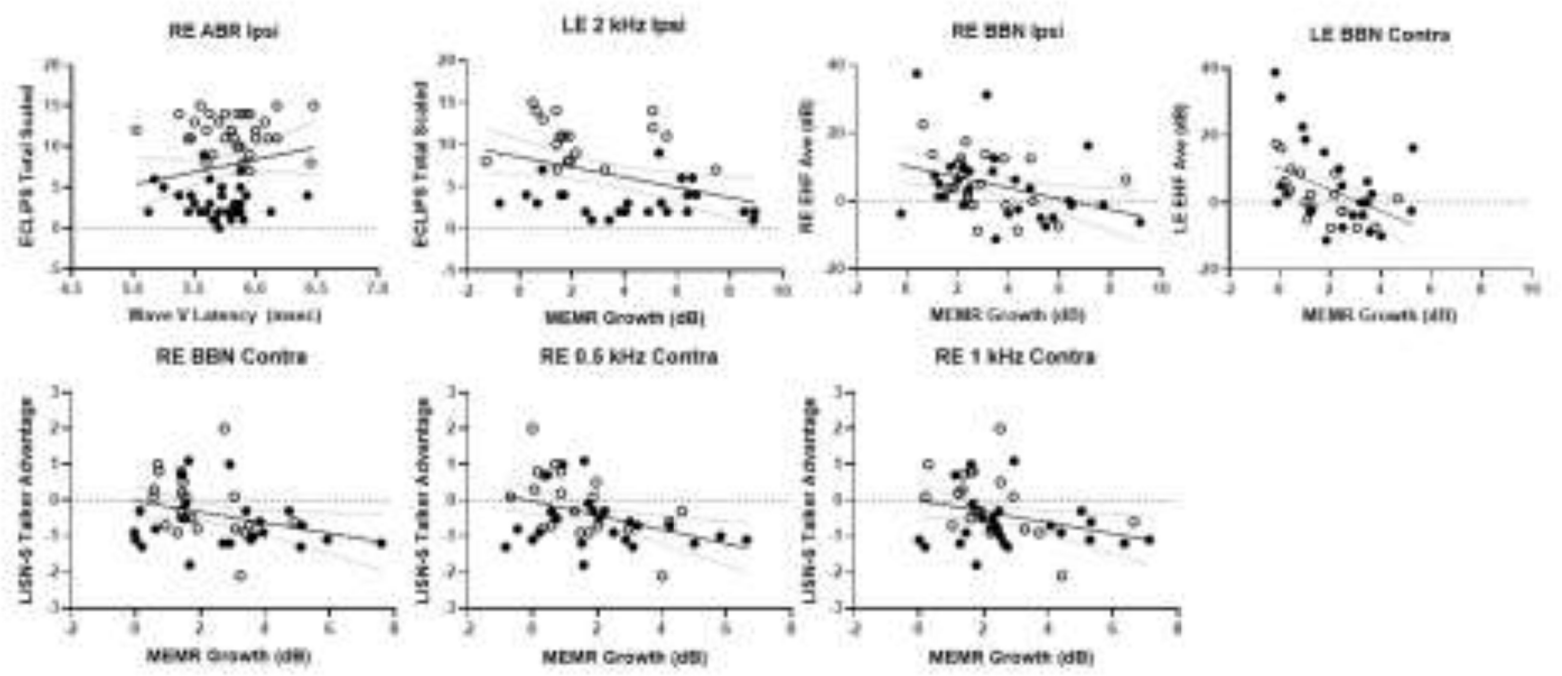
Correlations between ABR wave V latency and the ECLiPS total scaled score; between Average EHF hearing and MEMR growth; between MEMR left ipsilateral growth and the ECLiPS total scaled score; and between right contralateral MEMR growth and the LiSN-S talker advantage (in dB) for BBN, 0.5 kHz and 1 kHz stimuli. The LiD group (filled circle) and TD group (open circle) are shown separately, but the linear regression line and 90% confidence intervals are combined.

Due to the significant group differences for latency of ipsilateral ABR Waves III and V (RMANOVA), correlations with the Total ECLiPS score across all participants were examined using multiple forward stepwise regression. Shorter ipsilateral Wave V latency was correlated significantly with lower ECLiPS Total scores for the right ear (Figure 5A; r = −0.296, *p* = 0.023). Correlations for right ear Wave III and for left ear Waves III and V latency did not reach significance. Correlations between ABR Wave III or V with subtests of the LiSN-S test (low cue, high cue, talker, and spatial advantage scores) were not significant for either ear.

### 3.3 Middle Ear Muscle Reflex

**Figure 6.**
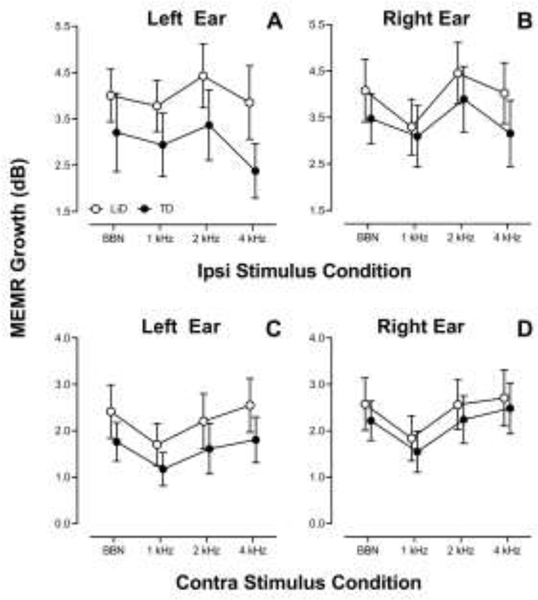
Wideband middle ear muscle reflex (MEMR) growth functions for ipsilateral (A, B) and contralateral (C, D) recording modes. The LiD and TD group are shown in open and filled circles, respectively. Error bars are 95% confidence intervals. N= 79 ears for the TD group and 87 ears for the LiD group.

MEMR responses were obtained from 54 LiD participants (n = 106 ears) and 49 TD participants (n = 95 ears). Ipsilateral and contralateral MEMR growth functions (amplitude growth as the stimulus level is increased) were examined with separate RMANOVAs. Stimulus type (BBN, 0.5, 1, and 2 kHz) was the repeated factor, group and ear were between subject factors, while age, sex, average EHF hearing levels (10–16 kHz) and tube history were included as covariates. Mean MEMR growth values, separated by laterality, stimulus type, and test ear, are shown in Figure 6 with the RMANOVA results in Table 3.

For ipsilateral MEMR growth, there was a significant effect of stimulus condition and group on MEMR absorbance with no other significant effects of sex, tube history, average EHF hearing level or age at test. The group effect size was small to medium (ƞ^2^_p_ = 0.038). For stimulus condition, post-hoc analysis showed that 1000 Hz resulted in a greater change in absorbance compared to 2000 Hz and 500 Hz with no significant difference compared to BBN or among the other stimulus frequencies (Figure 6; A, B). LiD participants showed greater MEMR growth, averaged across frequency compared to TD participants.

For contralateral MEMR growth, there was no significant effect of stimulus condition; however, there were significant effects of group, ear, sex, tube history and average EHF hearing level (Figure 6 C, D, Table 3). The group effect size was small to medium (ƞ^2^_p_ = 0.032). LiD participants showed greater MEMR growth compared to TD participants. The right ear showed a slightly greater MEMR growth compared to the left ear. Females displayed greater growth compared to males. Individuals with a history of tubes showed less MEMR growth compared to individuals with no tube history. Lastly, there was a significant effect of average EHF hearing level on the contralateral MEMR growth that was further explored in correlation analysis (below).

Correlations between MEMR growth and behavioral measures were examined across all participants using multiple forward stepwise regression for each “family” of tests to control for covariance (multiple stimulus frequencies within each MEMR test) and the ECLiPS standard score, and for each subtest of the LiSN-S test. There were significant negative correlations between EHF hearing and BBN MEMR growth functions, such that poorer EHF thresholds were related to lower MEMR growth (Figure 5). For the right ear, ipsilateral BBN MEMR was correlated with EHF hearing thresholds (r = −0.348, p = 0.013). For the left ear, contralateral BBN MEMR was correlated with EHF hearing thresholds (r = 0.449, p=0.003). There was a significant correlation between ipsilateral left ear MEMR growth at 2 kHz and ECLiPS scores, as shown in Figure 5 (r=−0.376, p=0.014). Correlations between contralateral right ear MEMR growth and the LiSN-S test talker advantage score were significant for BBN (Figure 5E; r=0.292, p=0.049) at 0.5 kHz (Figure 5; r=−0.403, p=0.006), and at 1.0 kHz (Figure 5; r=−0.354, p=0.016).

### 3.4 Binaural Medial Olivocochlear Reflex

**Figure 7.**
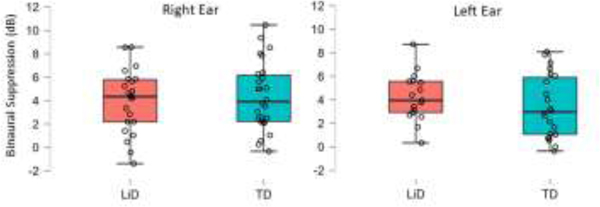
Box and stem overlaid with scatterplots for the binaural medial olivocochlear reflex (MOCR) expressed in dB of suppression compared to baseline response. The plots show median center line, interquartile range (box), 95% range (stems) and individual ears (open circles)Results for the left ear based on n=22 ears for the TD group and 17 ears for the LiD group (p=.314). Results for the right ear based on n=26 ears for the TD group and 22 ears for the LiD group (p=.636).

Binaural MOCR values are displayed as dB of suppression compared to the baseline response (Figure 7). Independent sample t-tests were conducted for the left and right ear separately to examine group differences. Results showed no significant MOCR group differences in the left (*p* = 0.636) or right ear (*p* = 0.314).

### 3.5 Envelope Following Response

**Figure 8.**
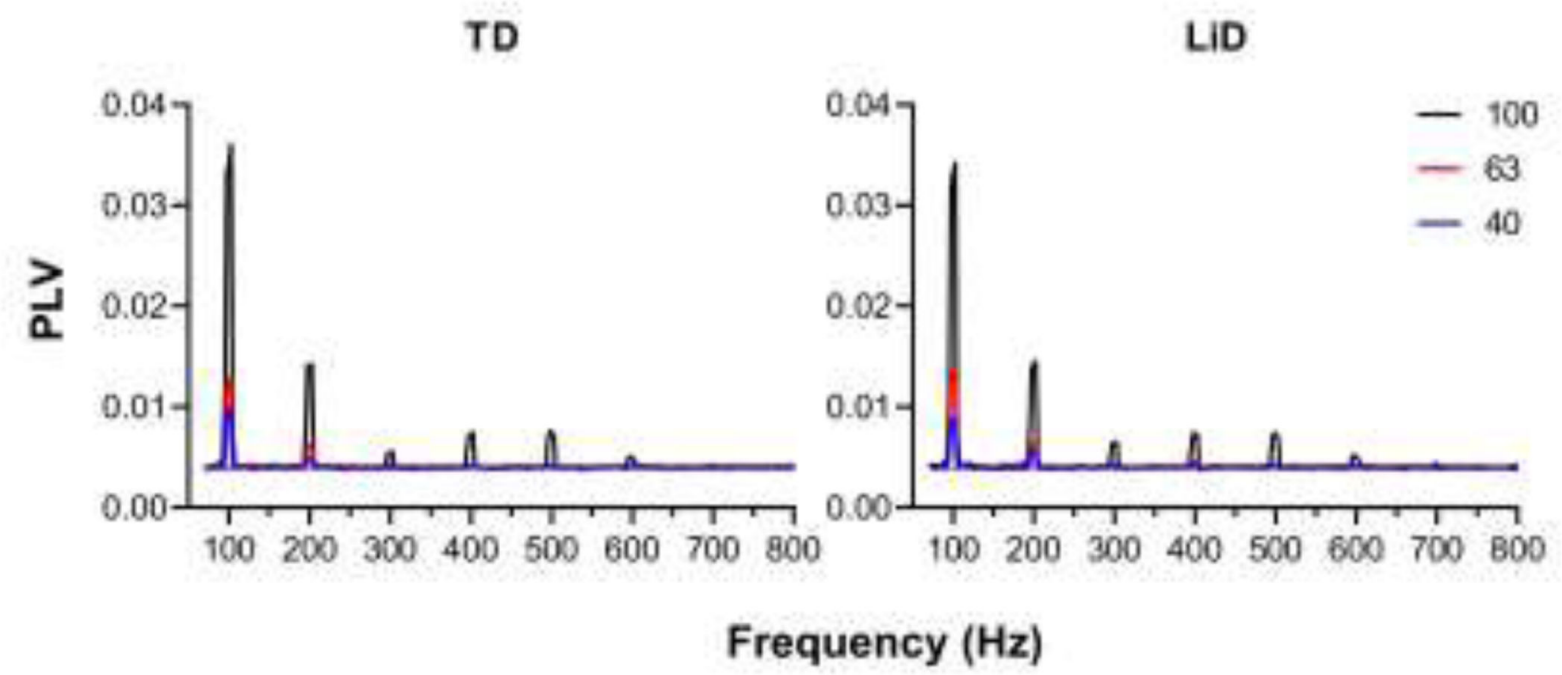
Average Phase Locking Value (PLV) as a function of frequency displayed for three modulation depths (100 = Black, 63 = Red, 40 = Blue). The TD group is shown in the left panel (N = 25 ears) and the LiD group (N = 30 ears) is shown in the right. Overall PLV analyzed by group p=0.851.

EFR amplitude and phase locking value (PLV) are displayed as a function of modulation depth for group averaged responses in Figure 8. Separate RMANOVAs were conducted for EFR amplitude, EFR SNR, PLV, PLV SNR with modulation depth (100%, 63%, and 40%) as the repeated condition (Table 4). Group was the between subject factor, and age, sex, average EHF hearing levels (10–16 kHz) and tube history were covariates.

No significant main group differences were found for EFR amplitude, PLV amplitude, EFR SNR and PLV SNR. There were no significant effects of sex, tube history, average EHF hearing level or age at test for the EFR or PLV measures. As expected, modulation depth EFR amplitude and SNR significantly increased with modulation depth, as did PLV SNR.

## 4.0 DISCUSSION

### Strengths and limitations.

The strengths of the study are that all test procedures were collected prospectively with an age-and sex-matched control group using the most sensitive and unbiased methods we could design. Blinding was applied for waveform interpretation, and the MEMR and MOCR procedures employed substantial controls to prevent observer bias, to control for noise and artifact, and applied automated detection algorithms rather than manual measurement. There are limitations as well, including a large age range, uncertainty of an APD diagnosis due to a lack of agreed-upon gold standard tests, differing referral patterns in different audiology clinics (that may mean differing underlying problems), and issues with noise and artifact for the ABR and MOCR tests that meant many subjects’ measures did not meet criteria for inclusion (although this was not different between LiD and TD groups).

### ABR and ECochG.

Shorter ABR Wave III and V latencies were found in the LiD group; these reduced latencies were related to poorer ECLiPS scores. The finding of shorter ABR wave III and V latencies for the LiD group was surprising, and in the opposite direction to that hypothesized, as delay in the ascending auditory pathway would logically relate to listening difficulties, with longer latencies reflecting slower speed of neuronal transmission. However, a previous study in 10 normal hearing children with learning disabilities and suspected APD compared to 10 age and gender matched control children reported significantly shorter Wave V latency and Wave III-V latency in the APD group (Purdy, Kelly, & Davies, 2002). The significant correlation between parent report of LiD and ABR Wave V latency has not been previously reported.

In contrast, previous studies of ABR latency in children with APD have mostly found normal results. In a study of children with suspected APD, with or without a defined diagnosis, ABR latencies and amplitudes were not related to diagnosis (Allen & Allan, 2014). A study of 20 children diagnosed with APD, tested with electrocochleography and ABR, found no significant absolute latency or interpeak latency group differences, compared to 16 typically developing children and 20 normal hearing adults (Veeranna, Allan, & Allen, 2021). Significantly smaller Wave V amplitudes were reported in the APD cases, but there was not a significant Wave V/I amplitude difference. A retrospective study of 108 children suspected of APD and tested with click ABR showed no significant group difference from typically developing children and adults, but some individual children (37%) were reported to have delayed latencies and less stable responses (Ankmnal-Veeranna et al., 2019). Similarly, a study of 19 children diagnosed with APD found no significant ABR latency or amplitude differences compared to 24 controls (Morlet et al., 2019).

### MEMR Growth Functions.

In a similar vein, if neural evidence for LiD were present in the lower brainstem, MEMR thresholds would be predicted to be higher (Saxena et al., 2017; Smart, Kuruvilla-Mathew, Kelly, & Purdy, 2019) and growth with stimulus level would be predicted to be shallower in children with LiD (Saxena et al., 2015). However, we previously found equivalent MEMR thresholds in LiD and TD groups, measured with wideband absorbance and real-ear calibration (Hunter et al., 2021). In the present study, we found significantly greater MEMR growth with increasing level in the LiD group for both ipsilateral and contralateral MEMR. Interestingly, we found significantly greater MEMR growth slopes in the left ear, compared to the right ear, but only in the LiD group. Of further interest is the relationship between poorer ECLiPS scores and greater ipsilateral left ear MEMR growth, and between poorer talker advantage on the LiSN-S and greater contralateral right MEMR growth. Regarding general trends in the data, the correlations across different stimuli were all in the same direction, but only some reached statistical significance in multivariate analyses since they were intercorrelated.

The steeper growth in the LiD group is difficult to reconcile with findings reported by Saxena and colleagues for threshold and growth of the MEMR. The wideband absorbance technique is designed to measure the MEMR over the frequency range of greatest activity, to control noise and produce a reliable response through signal averaging and automated detection algorithms and is reported to produce more sensitive thresholds by about 12 dB (Feeney & Keefe, 1999; Feeney et al., 2017). However, these differences would not be expected to reverse the direction of threshold differences or growth of the reflex. The most likely explanation based on our results is differing mechanisms for lower reflex thresholds and shallower growth curves that may reflect afferent or bottom-up effects, in contrast to increased reflex growth that more likely implicate efferent or top-down effects. (Blankenship et al., 2021; Hunter et al., 2021; Mishra, Saxena, & Rodrigo, 2022; Motlagh Zadeh et al., 2019). Examining the patterns across different stimuli and contralateral versus ipsilateral, it is clear that the TD and LiD groups differ across all stimuli for overall growth of the reflex both contralaterally and ipsilaterally, specifically in the left ear.

These effects in the LiD group were in the opposite direction from peripheral effects, where poorer EHF hearing was associated with shallower MEMR responses. In terms of peripheral hearing effects, two measures were related to poorer EHF hearing levels for both groups combined – longer ABR contralateral Wave V latency, and shallower MEMR growth and slope. Thus, some children in the LiD and TD groups showed a significant effect of poorer EHF hearing thresholds, despite having normal standard frequency thresholds. This finding may indicate diffuse cochlear deficits in EHF hearing loss despite normal hearing in the standard frequency range, as the stimuli used for ABR and MEMR are primarily below 4 kHz. Evidence has accumulated from recent reports that EHF hearing is related to speech in noise SRT (Flaherty, Libert, & Monson, 2021; Motlagh Zadeh et al., 2019; Polspoel, Kramer, van Dijk, & Smits, 2021) and to a range of physiologic measures in the standard frequency range. EHF threshold elevation is also an important early marker of peripheral auditory damage.

Evidence for adaptive plasticity measured with MEMR has been reported following short-term earplugging of one ear in adults, which induced a reduction in MEMR thresholds and an increase in perceived loudness (Brotherton, Plack, Schaette, & Munro, 2017), consistent with a compensatory increase in neural gain. In a related study, (Brotherton, Plack, Schaette, & Munro, 2016) found that ipsilateral and contralateral MEMR were decreased after 4 days of unilateral earplug use, but only when stimulation was applied to the plugged ear. These changes were consistent with a gain-control mechanism at the level of the ventral cochlear nucleus.

### EFR and PLV.

EFR and PLV measures have been proposed as sensitive to cochlear synaptopathy (Bharadwaj et al., 2014; Shaheen et al., 2015). We therefore included those measures in this study on the premise that CS may contribute to LiD. However, we found no evidence for the impaired temporal encoding that PLV is sensitive to in the LiD group.

### MOCR.

The binaural MOCR paradigm we used has been previously shown to produce the largest and most reliable effect of the different MOCR methods (Lilaonitkul & Guinan, 2009). Despite rigorous quality control measures for attention, SNR, sufficient averaging, and control of the MEMR threshold, only 38% of MOCR recordings overall met quality criteria to be included in the analysis. This is a significant limitation of these results, so interpretation must be cautious with respect to an absence of group significance. Test-retest reliability was poorer in the LiD group and may be related to poorer attention (Moore, Ferguson, Edmondson-Jones, Ratib, & Riley, 2010; Stavrinos, Iliadou, Edwards, Sirimanna, & Bamiou, 2018) and greater internal noise (Porter, Leibold, & Buss, 2020) in that group. It may also be the case that the MOCR as measured by the TEOAE suppression method is not related to difficulties in natural listening situations, consistent with some previous studies (Mattsson et al., 2019; Smart et al., 2019).

A recent investigation by Rao, Koerner, Madsen, and Zhang (2020) in adults reported no significant effect of contralateral MOCR activation on auditory cortical responses or on a nonsense word recognition in noise task. They concluded that the MOCR may not play a primary role in higher level processing of speech in noise perception. Related to this hypothesis, Hernandez-Perez et al. (2021) measured MOCR strength using TEOAE suppression and compared it to neural activity along the ascending pathways in response to “degraded” (vocoded) or conventional noise-masked speech. The MOCR was activated by the vocoded speech signal, but not by speech-in-noise which, instead, increased neural activity in the midbrain and cortex. They suggested that the auditory system has distinct strategies to manage these two types of distorted speech. In the ear and hindbrain, the MOCR may enhance the stimulus waveform, whereas the mid- and forebrain may be specialized to reduce the effect of added noise.

### Overall patterns and potential mechanisms.

There are two different classes of efferent pathways, MOCR and MEMR, that could relate to listening difficulties, despite normal hearing thresholds. They arise from different mechanisms, and have different actions, on the outer hair cells in the case of the MOCR and on the stapedius muscle in the case of the MEMR. They are measured with different techniques, and the MEMR effect is much larger than the MOCR effect. There are therefore many reasons that one type of efferent response may be affected while the other is not. Our finding of no significant differences between groups for some auditory neural measures (MOCR, EFR, SP and Wave I) is consistent with the hypothesis that lower-level auditory pathway function does not contribute to the LiD problems experienced by children in this study. Group differences emerged, however, for pathways involved in ABR Waves III and V, and MEMR growth, in the region of the cochlear nucleus. Poorer subcortical control and leftright balance of auditory information was indexed by 1) shorter Wave III and V latencies, with right Wave V related to poorer caregiver reports of LiD, and 2) steeper MEMR growth, related to performance in a natural, sentence-in-distractor task and the validated parent reports of LiD. These findings suggest the possibility that children with LiD may have atypical corticofugal mechanisms. Poorer corticofugal control is consistent with a range of other evidence for poorer cortical function in children with LiD (Dawes & Bishop, 2009; Ferguson, Hall, Riley, & Moore, 2011; Miller & Wagstaff, 2011; Moore, Ferguson, Edmondson-Jones, Ratib, & Riley, 2010; Petley et al., 2021b; Sharma, Purdy, & Kelly, 2009).

As reviewed by Souffi et al. (2021), recent animal evidence suggests that, during active listening, the frontal cortex activates the auditory cortex (AC) and IC directly and, via the AC, the MGB and IC indirectly, producing strong top-down control. Because the ABR and MEMR measures we employed here did not involve active listening to natural speech in noise, it seems unlikely that such downward control mechanisms were operational. Rather, the shorter latency and steeper growth curves in the LiD group may reflect increased central gain (Souffi et al., 2021) having further downstream influences on brainstem ABR Waves III and V, and the MEMR. We are currently in the process of analyzing active speech-evoked and passive resting state MRI in these same children and will evaluate this hypothesis regarding functional connectivity of the frontal regions, primary auditory cortex, and auditory thalamus.

## 5.0 CONCLUSIONS

In conclusion, brainstem-based enhancements of ABR Waves III and V, and MEMR growth, were negatively correlated with parent report of listening ability and perception of speech sentences in competing speech.. In contrast, broad band MEMR growth was positively correlated with better EHF sensitivity, and neural phase locking was independent of listening ability, suggesting that peripheral function did not account for the brainstem results. Surprisingly, children with listening difficulties thus had faster brainstem processing and larger MEMR reflexes than their typically developing peers. These results may be interpreted in a framework of enhanced, cortically-mediated efferent function as a mechanism for listening difficulties. However, effect sizes were small, and further investigation of this hypothesis is needed.

## Figures and Tables

**Table 1.** Study demographics for all participants, the typically developing (TD) group, and the listening difficulty group (LiD) group.

	All	TD	LiD	*p*-value
**Number of Participants** ^a^	132	69	63	−
**Age (yrs.)** ^a^				0.599 ^d^
Mean (SD)	10.0 (2.1)	9.9 (2.1)	10.1 (2.1)	
Range	6.2–14.6	6.3–14.6	6.2–14.1	
**Sex** ^a^				0.631 ^e^
Male	81 (61.4%)	41 (59.4%)	40 (63.5%)	
Female	51 (38.6%)	28 (40.6%)	23 (36.5%)	
**Race** ^a^				0.172 ^e^
Caucasian	107 (81.1%)	59 (85.5%)	48 (76.2%)	
Non-Caucasian	25 (18.9%)	10 (14.5%)	15 (23.8%)	
**Ethnicity** ^a^				0.550 ^e^
Hispanic or Latino	5 (3.8%)	2 (2.9%)	3 (4.8%)	
Non-Hispanic or Latino	125 (94.7%)	67 (97.1%)	58 (92.0%)	
Prefer Not to Answer	2 (1.5%)	0 (0%)	2 (3.2%)	
**Maternal Education Level** ^a^				***0.001*** ^e^
Graduated high school or less	12 (9.1%)	1 (1.4%)	11 (17.5%)	
Some college or more	120 (90.9%)	68 (98.6%)	52 (82.5%)	
**PE Tubes** ^a^				0.605 ^e^
No History of Tubes	100 (75.8%)	51 (73.9%)	49 (77.8%)	
History of Tubes	32 (24.2%)	18 (26.1%)	14 (22.2%)	
**EHF Hearing Status** ^b^				***0.033*** ^e^
Normal	184 (74.5%)	96 (73.8%)	88 (75.2%)	
Hearing Loss	39 (15.8%)	13 (10.0%)	26 (22.2%)	
Not Measured	24 (9.7%)	21 (16.2%)	3 (2.6%)	
**ECLiPS** ^c^				
Scaled Score	7.1 (4.5)	10.8 (2.5)	2.9 (1.7)	***<0.001*** ^d^
**LiSN-S** ^c^				
Low-Cue	−0.5 (1.7)	−0.1 (1.0)	−1.0 (2.1)	***<0.001*** ^d^
High-Cue	0.01 (1.2)	0.4 (1.0)	−0.4 (2.1)	***<0.001*** ^d^
Talker Advantage	−0.3 (0.9)	−0.04 (0.8)	−0.5 (1.0)	***<0.001*** ^d^
Spatial Advantage	−0.3 (1.5)	−0.1 (1.2)	−0.6 (1.7)	***0.002*** ^d^
Total Advantage	0.3 (1.2)	0.5 (1.0)	0.1 (1.3)	***0.002*** ^d^

Note:

a = number (%) of participants;

b = number (%) of ears;

c = mean (SD);

d = Two-Sample t-test;

e = Chi-Square test;

Bold italics indicate significant *p*-values.

**Table 2.** ABR repeated measures analysis of variance *p*-and F-values for factors included in the final model.

	Intensity	Group	Ear	Sex	Tube History	EHF HL	Age
**Ipsilateral**							
SP Latency (p)	***<.030***	0.439	***0.018***	0.932	0.725	0.219	0.967
F (DF = 1 to 3.0)	***3.049***	0.603	***5.792***	0.007	0.125	1.531	0.002
Wave I Latency (p)	***<.001***	0.708	***0.047***	0.946	0.988	0.122	0.376
F (DF = 1 to 2.5)	***10.833***	0.141	***4.043***	.005	<0.001	2.434	0.791
Wave III Latency (p)	0.168	***0.001*** *	0.582	0.227	0.139	0.409	0.877
F (DF = 1 to 2.5)	1.738	***10.714***	0.301	1.475	2.216	0.688	0.024
Wave V Latency (p)	***.002***	***0.029*** **	0.848	***0.026***	0.687	0.171	0.206
F (DF = 1 to 2.3)	***5.727***	***4.887***	0.037	***5.072***	0.163	1.899	1.620
SP Amplitude (p)	***<.001***	0.358	0.071	***0.042***	0.130	0.271	***0.045***
F (DF = 1 to 3.0)	***5.502***	0.393	2.886	***3.011***	2.336	1.228	***4.150***
Wave I Amplitude (p)	***0.018***	0.200	0.129	***0.001***	0.079	0.293	0.036
F (DF = 1 to 2.6)	***3.651***	1.663	2.335	***10.618***	3.144	1.118	4.520
Wave III Amplitude (p)	0.633	0.991	CNT	0.265	0.182	0.252	0.482
F (DF = 1 to 3.0)	5.766	0.001	CNT	1.321	1.931	1.401	0.516
Wave V Amplitude (p)	0.552	0.307	0.302	***0.011***	0.439	0.439	0.590
F (DF = 1 to 2.6)	5.32	1.055	1.075	***6.685***	0.604	0.604	0.292
Wave V/I Amplitude (p)	0.655	0.930	0.092	0.742	0.870	0.724	0.098
F (DF = 1 to 2.6)	0.446	0.008	2.898	0.109	0.027	0.125	2.792
**Interpeak Latencies**							
I-III, III-V, I-V Latency (p)	NA	0.087	0.342	***0.027***	0.722	0.453	0.463
F (DF = 1)		2.984	0.909	***5.010***	0.127	0.566	0.542
**Contralateral**							
Wave V Latency (p)	0.205	0.089	0.910	0.112	0.346	***0.029***	0.424
F (DF = 1 to 2.4)	1.538	2.960	0.013	2.577	0.896	***4.929***	0.646
Wave V Amplitude (p)	0.591	0.408	***0.035***	0.740	0.347	0.522	0.506
F (DF = 1 to 3.0)	0.570	0.697	***4.690***	0.111	0.901	0.416	0.450

*Note:* CNT = Could not test; DF = Degrees of Freedom; EHFHL = Extended High Frequency Hearing Loss (> 20 dB HL, 10 to 16 kHz); Bold italics indicate significant p-values.

* Effect size η^2^_p_ = 0.091

** Effect size η^2^_p_ = 0.04

**Table 3.** MEMR growth repeated measures analysis of variance, with *p*-values and F-test (Degrees of Freedom) for factors included in the final model.

	Stimulus Type	Group	Ear	Sex	Tube History	EHF HL	Age
**Ipsilateral**							
MEMR Growth	***0.034***	***0.011*** *	0.260	0.066	0.064	0.109	0.555
F (DF = 1 to 2.8)	***3.000***	***6.625***	1.277	3.426	3.476	2.592	0.350
**Contralateral**							
MEMR Growth	0.086	***0.019*** **	***0.015***	***0.005***	***0.039***	***<.001***	0.374
F (DF = 1 to 2.8)	2.254	***5.587***	***5.991***	***8.160***	***4.340***	***16.448***	0.796

*Note:* Stimulus Condition = BBN, 0.5, 1, 2 kHz; EHFHL = Extended High Frequency Hearing Loss (> 20 dB HL 10 to 16 kHz) from; Bold italics indicate significant p-values.

* Effect size η^2^_p_ = 0.038

** Effect size η^2^_p_ = 0.032

**Table 4.** EFR repeated measures analysis of variance, with *p*-values and F-test (Degrees of Freedom) for factors included in the final model.

	Modulation Depth	Group	Sex	Tube History	EHF HL	Age
**EFR**						
Amplitude (p)	***0.002***	0.186	0.221	0.257	0.359	0.057
F (DF = 1 to 1.1)	***9.843***	1.812	1.544	1.326	0.863	3.834
SNR (p)	***0.021***	0.482	0.317	0.862	0.385	0.475
F (DF = 1 to 1.6)	***4.474***	0.503	1.024	0.031	0.771	0.520
**Phase Locking**						
Value (p)	0.179	0.851	0.909	0.373	0.270	0.867
F (DF = 1 to 1.1)	1.868	0.036	0.013	0.810	1.251	0.028
SNR (p)	***0.019***	0.669	0.821	0.521	0.513	0.548
F (DF = 1 to 1.7)	***4.526***	0.185	0.052	0.419	0.434	0.367

*Note:* EHFHL = Extended High Frequency Hearing Loss (> 20 dB HL, 10 – 16 kHz); Bold italics indicate significant p-values.

## Highlights

- Auditory brainstem responses had shorter latency Wave III and V in LiD.
- Middle ear muscle reflexes had increased growth in children with LiD.
- ABR and MEMR related to reported listening problems and speech in noise.

## ABBREVIATIONS

ABR: Auditory Brainstem Response
APD: Auditory Processing Disorder
BBN: Broadband Noise
CCH: Cincinnati Children’s Hospital
ECLiPS: Evaluation of Children’s Listening and Processing Skills
ECochG: lectrocochleography
EFR: Envelope Following Response
EHF: Extended high frequency
LiSN-S: Listening in Spatialized Noise - Sentences
LiD: Listening Difficulty
MOCR: Medial Olivocochlear Reflex
MEMR: Middle Ear Muscle Reflex
OAE: Otoacoustic Emission
SP: Summating Potential
TEOAE: Transient Evoked Otoacoustic Emission
TD: Typically Developing
